# Spatio‐Temporal Decoding of the Navon Task Challenges Rigid Hemispheric Asymmetries in Global–Local Processing

**DOI:** 10.1111/psyp.70032

**Published:** 2025-03-04

**Authors:** Tobias Hausinger, Patrick Reisinger, Nathan Weisz, Andrea Hansen, Ti‐Anni Harris, Belinda Pletzer

**Affiliations:** ^1^ Centre for Cognitive Neuroscience University of Salzburg Salzburg Austria; ^2^ Department of Psychology University of Salzburg Salzburg Austria; ^3^ Neuroscience Institute, Christian Doppler University Hospital Paracelsus Medical University Salzburg Salzburg Austria

## Abstract

Functional hemispheric asymmetries are considered a key factor in intra‐ and interindividual variability of global precedence effects. However, research in this area is permeated by a considerable number of inconsistent findings which may stem from significant methodological limitations. In pursuit of a more detailed model of global–local processing by combining both high temporal and spatial resolution, we employed Multivariate Pattern Analysis (MVPA) on Magnetoencephalography (MEG) recordings from 63 participants performing a divided visual field, divided attention Navon paradigm. The resulting decoding accuracies between various hierarchical letter forms and target levels were used to pinpoint potentially involved spatial networks and temporal processing sequences. Linear Discriminant Analysis (LDA) revealed temporal precedence of global over local letter form decoding accuracy peaks. Furthermore, searchlight analysis provided a nuanced spatial mapping that not only validated previously established core regions (lingual gyrus for local processing; inferior occipital gyrus for global processing) but also identified potential regions implicated in global–local integration. Yet, we observed substantial variation in lateralization patterns across our study sample, challenging the conventional assumption of right‐hemispheric dominance for global and left‐hemispheric dominance for local processing in the context of MVPA. Overall, our findings validate and broaden the scope of prior research by providing, for the first time, accurate temporal and spatial data on global–local processing from a single measurement. Moreover, we introduce interindividual variability in lateralization patterns as a potential factor contributing to past inconsistencies.

## Graphical abstract

Research on global‐local processing and associated functional hemispheric asymmetries (FHAs) provides an important theoretical framework for understanding mechanisms of attention, perception, and memory. Utilizing Multivariate Pattern Analysis (MVPA) of Magnetoencephalography (MEG) recordings, we provide a complementary, fine‐grained overview of global‐local processing steps, featuring an unprecedented combination of temporal and spatial resolution. The discriminative power of MVPA yielded novel insights into distinct networks involved in each processing stage and revealed significant inter‐individual variation in FHAs as a potential factor to consider in future research.

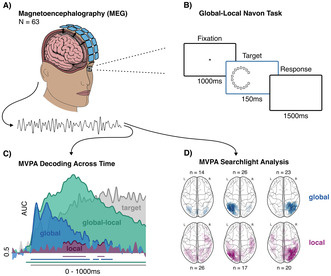

## 1 Introduction

Pattern recognition research in cognitive neuroscience provides consistent evidence in favor of global precedence, i.e., faster processing of global configurations compared to their local elements (see Kimchi 2014 for a review). It is commonly assumed that the right hemisphere shows a stronger bias toward global‐level processing, whereas the left hemisphere has an advantage in local‐level processing (Karim and Kojima 2010) or at least a similar specialization for both levels (Martinez et al. 1997).

Neuropsychological evidence revealed impaired responses to local details of a visual scene in patients with left‐hemispheric lesions, while responses to global configurations were similarly impaired in those with right‐hemispheric lesions (Delis et al. 1988; Robertson and Lamb 1991; Robertson et al. 1988). In neurotypical populations, the so‐called global advantage effect (GA), that is, faster and/or more accurate responses to global compared to local stimulus aspects, was generally more pronounced when stimuli were presented in the left visual field and thus initially processed in the right hemisphere (LVF‐RH) than when they were presented in the right visual field (RVF‐LH) (Hausinger and Pletzer 2021; Kimchi and Merhav 1991; Sergent 1982).

However, dedicated electrophysiological and neuroimaging studies confirming global–local processing asymmetries are scarce. During positron emission tomography (PET), relative cerebral blood flow (rCBF) in the right lingual gyrus (LG) was increased in tasks requiring attention to the global stimulus level, while the highest rCBF levels for attention to the local stimulus level were located within the left inferior occipital gyrus (IOG) (Fink et al. 1996; Fink, Halligan, et al. 1997). Similarly, performance in global or local level processing was enhanced with either cued or TMS‐induced alpha‐band activity decreases in the specialized hemisphere and alpha‐band increases in the non‐specialized hemisphere (Romei et al. 2012; Volberg et al. 2009).

Yet, the idea of a generally accepted global LVF‐RH and local RVF‐LH asymmetry model faces objections, as a considerable number of studies have identified the implied pattern exclusively at one level (Alivisatos and Wilding 1982; Hubner 1997; Martin 1979), in reverse (Fink, Marshall, et al. 1997; Keita and Bedoin 2011), or not at all (Boles 1984; Mena 1992; Polich and Aguilar 1990; Van Kleeck 1989). The exact mechanisms determining FHAs appear highly complex, as factors such as stimulus position, spatial frequency (Han et al. 2002; Lamb and Robertson 1988) and verbal content (Fink, Marshall, et al. 1997; Polich and Aguilar 1990), as well as various task aspects including attention condition (Yovel et al. 2001) have emerged as powerful asymmetry modulators. More accurate insights into the specific processing stages at which hemispheric asymmetries may or may not occur could thus help develop more reliable models of global–local processing and support efforts to clarify past inconsistencies.

Most of the information on global–local processing sources, their lateralization, and temporal dynamics relies on sensor‐level event‐related potential (ERP) analyses of electroencephalography (EEG) data. Accordingly, signal asymmetries between electrodes over contralateral, occipito‐parietal electrodes occurred as early as 100 ms after stimulus onset for selective attention paradigms (Evans et al. 2000), while others were not able to detect lateralization until 200–350 ms (Heinze and Munte 1993) for divided attention paradigms. Yet, such previous ERP‐based approaches implicitly make the invalid inference of anatomical information (i.e., source locations) from mere sensor positions. However, recorded surface‐level signals at any recording electrode represent a mixture of numerous potential source configurations throughout the brain. Moreover, standard template electrode positions in previous studies may deviate substantially from subject‐specific areas (Ignatiadis et al. 2022). To mitigate these issues, attempts at source reconstruction should be applied to reach more reliable conclusions regarding hemispheric asymmetries.

Despite offering valuable insights into the structural and temporal properties of global–local processing, prior investigations were not geared toward combining both domains simultaneously. Although the current experiment does not manipulate all of the aforementioned factors influencing hemispheric asymmetry, such as stimulus properties or attention condition, we aim to suggest a methodologically comprehensive spatio‐temporal framework of global–local processing as a starting point for future studies to complete and build upon. We do so by utilizing both high temporal and comparatively accurate spatial resolution of magnetoencephalography (MEG) in combination with multivariate pattern analysis (MVPA). In contrast to EEG, our current approach offers the advantage that anisotropic conductivities of tissues do not limit the spatial resolution of MEG, allowing for a more accurate source localization of brain activity. Moreover, the use of MVPA in particular provides unique insights into the global–local processing hierarchy.

Using a hierarchical letter Navon paradigm, where large (global) letters are composed of smaller (local) letters, we train a classifier to differentiate brain activity associated with different letter shapes at the same hierarchical level or with compound letters containing a target at either the global or local level. This allows us to identify time windows and regions that may be related to various processing steps, ranging from lower‐level perceptual differentiation to higher‐level information integration. In addition, MVPA's incorporation of electrophysiological characteristics, including signal frequency, amplitude, and phase, enhances sensitivity in identifying specialized brain regions that may remain unnoticed with previously employed methodologies. We aim to: (1) outline a potential chronology of lower‐and higher‐level processes; (2) identify a network of brain regions contributing the most informative activity during each step; (3) pinpoint hemispheric asymmetries within global–local processing networks.

## 2 Methods

### 2.1 Participants

Data from 63 participants were included in the analysis (27 male, 36 female, age range = 18–35 years, mean age = 24.5 ± 4.1 years). All participants were right‐handed, reported normal or corrected‐to‐normal vision, and confirmed that they had not been diagnosed with psychological, neurological, or endocrinological disorders. Participants provided written informed consent before the experiment and were credited with 10€/h compensation or ECTS credits required for bachelor studies. All procedures were approved by the Ethics Committee of the University of Salzburg and adhered to the Declaration of Helsinki (2014).

### 2.2 Stimuli and Procedure

An approximate total of 300 head‐shape points, three anatomical landmarks (nasion, left and right pre‐auricular points) and five head‐position indicator coils (three on forehead, one behind each ear) were specified with a Polhemus FASTRAK digitizer (Polhemus, Colchester, Vermont, USA). To further control for physiological artifacts, two additional electrodes recorded electrooculograms and electrocardiograms, respectively.

**FIGURE 1:**
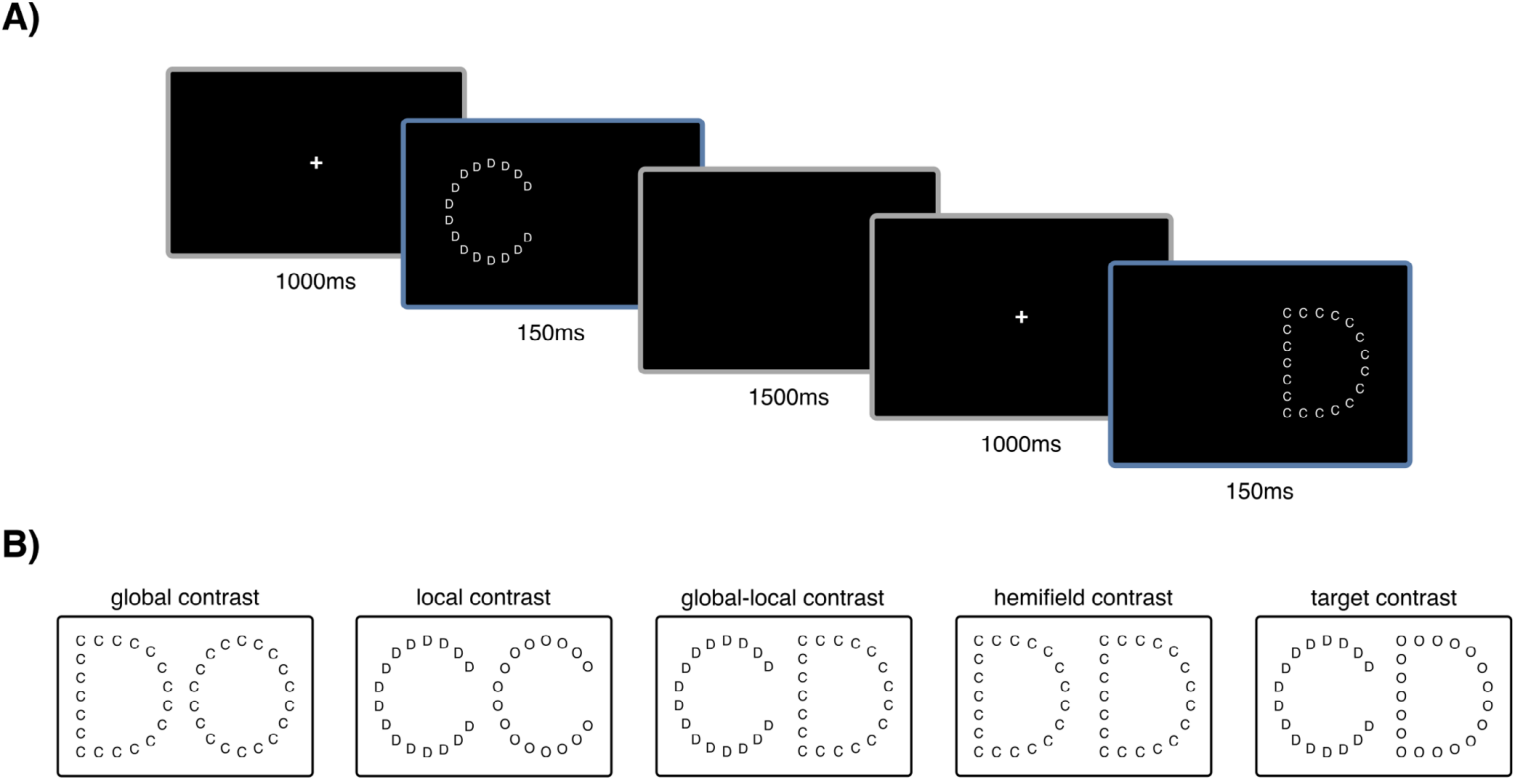
(A) Trial structure: Each trial lasted a maximum of 2550 ms, starting with 1000 ms of central fixation, followed by 150 ms left or right hemifield stimulus presentation and a blank screen lasting until response or for a maximum of 1500 ms. (B) Examples of stimulus contrasts fed into the classifier with target letter “C”. Global contrasts consistently used the same local target letter, whereas local contrasts consistently used the same global target letter.

First, a 5‐minute resting‐state recording was performed. In the subsequent main experiment, global–local processing was assessed with classic hierarchical letter stimuli (Navon 1977) in two different hemifield conditions (left visual field—right hemisphere: LFV‐RH, right visual field—left hemisphere: RFV‐LH). Stimuli were constructed from the letters C, D, and O, resulting in six distinct hierarchical letter combinations Co, Cd, Dc, Do, Od, Oc (uppercase letters representing global letters). The distance between local letters (approximately 7% of the global letter size) was comparable to local letter size (approximately 10% of global letter size). Each trial started with 1000 ms of central fixation, followed by 150 ms stimulus presentation and a blank screen until response or a maximum of 1500 ms before initiating the next trial (Figure 1A). Participants were assigned one random target letter for the whole duration of the recording session and instructed to identify the target at any hierarchical level (global or local, i.e., divided attention condition) by pressing a button (left = target present, right = no target present) on a response box with their dominant (right) hand. A total of 900 trials (300 global targets, 300 local targets, 300 non‐targets) were performed, separated into three blocks with 100 targets of each category per block. Stimuli were displayed with MATLAB R2016b (The Math‐Works, Natick, Massachusetts, USA) via rear projection (PROPixx DLP projector, VPixx technologies, Canada) to a 30‐in. translucent screen with a resolution of 1920 × 1080 pixels at a viewing distance of ~110 cm.

Global letters subtended a visual angle of 5.42° × 6.16° (301 × 342 px), while local letters subtended a visual angle of 0.50° × 0.63° (28 × 35 px). Half of the trials (150 global, 150 local, and 150 non‐targets) were presented in each visual hemifield, respectively, with the center of each global stimulus form deviating 510 px along the x‐axis from the center of the screen.

Fixation control was implemented by instructing participants to attend to a central fixation cross. While no eye‐tracking was used and initial fixation accuracy was not controlled for, stimulus presentation time was less than 200 ms, ruling out the possibility of saccadic eye movements towards the stimulus. As hemifield presentation was randomized, strategies involving initial gaze deviations from the center should not have resulted in above‐chance response accuracy levels.

### 2.3 Decoding Contrasts

Five different hierarchical letter contrasts were investigated with the MVPA approach (Figure 1B). We compared decoding performance for different global—same local letter combinations (global contrast), same global—different local letter combinations (local contrast), global and local target letters (global–local contrast), right and left hemifield letters (hemifield contrast), as well as target and non‐target letters. Global and local contrasts always consisted of target letters at the level that was irrelevant to the contrast. The hemifield of presentation was only considered for the hemifield contrast.

### 2.4 MEG Acquisition

Magnetic brain signals were recorded at a sampling rate of 1 kHz at 102 magnetometers and 204 orthogonal planar gradiometers covering 102 distinct positions in a whole‐head MEG system (Elekta Neuromag Triux, Elekta Oy, Finland). Hardware filtering was applied from 0.1 to 330 Hz. Recordings were obtained inside a passive magnetically shielded room (AK3b, Vacuumschmelze, Germany).

### 2.5 Preprocessing

A signal space separation algorithm (Taulu et al. 2005) implemented in Maxfilter (version 2.2.15, Elekta) was applied to remove external noise, such as power line noise (50 Hz) and nearby train artifacts (16.7 Hz) from the MEG signal while preserving data integrity. Signal space separation also compensates for head movements by realigning head positions across recording sessions to a common standard head position (to [0 0 40] mm, −trans default Maxfilter parameter), improving data comparability and enhancing the signal‐to‐noise ratio (SNR). MaxFilter further reduces noise by automatically detecting and interpolating bad channels.

Following MaxFilter application, continuous data was passed through a high‐pass filter at 0.5 Hz (6th order zero‐phase Butterworth) and epoched around stimulus onset with a 0.5‐s pre‐ and 1‐s post‐stimulus window. Second, independent component analysis (ICA) was applied using the runica algorithm (Delorme and Makeig 2004) and components were visually inspected and removed if associated with eye blinks, eye movements, heartbeat, or train artifacts (≈ 3–5 components per recording). After component removal, individual trials from each recording were inspected for any remaining outliers or extreme noise. Due to extensive preprocessing, no trials had to be rejected. Data was downsampled to 100 Hz for subsequent analyses.

### 2.6 Source Projection of MEG‐Data

To ensure accurate spatial mapping, participants' head shapes were coregistered with their individual structural MRIs using fiducials as reference landmarks. The coregistration results were then manually inspected for optimal alignment. A 3‐D MNI template grid with 8 mm resolution and 2205 cortical gray matter voxels based on a standard structural template brain (MNI, Montreal, Canada) was morphed to individual brain volumes, allowing for group‐level averaging and statistical analysis across subjects. Aligned volumes were used for single‐shell head model and leadfield computation (Nolte 2003). Respective leadfields and a common covariance matrix were applied for LCMV beamformer spatial filter computation (Van Veen et al. 1997). Quality control of source reconstruction involved manual inspection of visual cortex responses to single hemifield presentation stimulus groups, ensuring accuracy in both lateralization and cognitive domain mapping. The obtained decoding accuracy of ~96% for hemifield presentation further validates source integrity.

### 2.7 MVPA Analysis

Cross‐condition decoding analysis was conducted in two steps. First, LDA classifiers were trained and tested on whole‐brain sensor data to define 150‐ms time windows of interest including respective area under the curve (AUC) peaks. LDA classifiers were applied to a time window of 0 to 1000 ms from stimulus presentation with standard settings of the MVPA toolbox, including a fivefold cross‐validation scheme (training on 80% of trials, testing on 20% of trials), stratified, repeated five times, and finally averaging AUC values across fold splits and repetitions to obtain the reported decoding performance (M.S. Treder 2018). AUC was estimated using the Mann–Whitney U statistic as implemented in the MVPA‐Light toolbox's mv_calculate_performance function. This method calculates the probability that a classifier can correctly recognize that a randomly chosen sample from one class (e.g., global target letters) has different underlying activity patterns than a sample from the other class (e.g., local target letters) by directly comparing their classifier decision values without constructing the ROC curve. This approach provides an efficient and direct estimation of the AUC. Decoding accuracies were tested against chance level (AUC = 0.5) between 0 and 1000 ms after stimulus onset using a non‐parametric cluster‐based permutation approach (Maris and Oostenveld 2007) with 10,000 randomizations.

Second, resulting time windows were used for source‐space searchlight analysis to identify relevant regions contributing the most informative activity, using each of the 2205 virtual channels, its three closest neighbors, as well as all 15 × 10 ms time points per channel of the 150 ms time windows as features for cross‐validation. Cross‐validation was implemented with five folds and 100 repetitions. Channel accuracies were then tested against chance level using a MC‐corrected cluster‐based permutation approach.

All cluster‐based permutations were computed with Monte Carlo randomization across grid points (searchlight) or time points (sensor‐ and ROI‐decoding) controlling for multiple comparisons. All permutation tests were conducted using MATLAB R2020a (The MathWorks, Natick, Massachusetts, USA). All data preprocessing, sensor, and source analyses were performed with the Fieldtrip toolbox (Oostenveld et al. 2011). For the decoding analysis, the MATLAB‐based open‐source MVPA‐Light toolbox (M. S. Treder 2020) was used.

### 2.8 Asymmetry Analysis

*t*‐values from global‐ and local‐letter searchlight analyses were used for asymmetry analysis, respectively. Regions of interest (ROIs) from the Brainnetome atlas (Fan et al. 2016) were included based on a data‐driven *t*‐value criterion. A region was included in the asymmetry analysis whenever the average *t*‐value of all its voxels reached at least 80% of the maximum *t*‐value region. Consequently, *t*‐values of all ROI channels were compared between hemispheres using a Bayesian independent t‐test approach to determine lateralization. Since the lateralization of global and local searchlight contrasts was heterogeneous across individuals (compare Section 3.3), we also used each individual's AUC lateralization distribution across the employed 100 cross‐validation repetitions as a reliability measure for the observed individual lateralization profile. General consistency across different training‐ and test‐trial subsets was furthermore assessed by computing an Interclass Correlation Coefficient (ICC) among all 100x2205x1 result vectors for each subject. Similarly, to identify asymmetric regions during each processing step, the absolute hemispheric differences (HA) of each ROI's mean accuracy values across the sample were tested against zero. t‐tests were performed in R using the “BayesFactor” package (Morey and Rouder 2018) (version 0.9.2) with noninformative Jeffreys and Cauchy priors.

### 2.9 Behavioral Analysis

To test whether reactions to global targets were significantly faster than reactions to local targets, the global advantage effect (GA) was calculated for each participant as a standardized contrast (Zhang 2010), i.e., the difference in mean reaction times between local and global targets divided by the standard deviation. In the following group analysis, one‐sample t‐tests were performed comparing GA to zero. One subject was excluded from the behavioral analysis since response accuracy was below chance level.

To test for relationships between AUC decoding accuracies and behavioral measures, several linear mixed‐effects models were fitted to the data using the lmer function of the lme4 package (Bates et al. 2015) in R version 4.1.2, entering GA as the dependent variable, participant number (PNr) as a random intercept, and AUC decoding accuracies or hemispheric decoding asymmetry scores (HA) as fixed factors alongside the visual hemifield.

For accuracies, ROI‐based AUC, hierarchical target letter level, visual field of presentation, and all two‐ and three‐way interactions were entered as fixed factors [model (1): GA ~ 1 | PNr + AUC * visualfield].

For hemispheric asymmetries, an absolute difference score (HA) between decoding accuracies of each identified ROI and its contralateral counterpart was calculated, and alongside the visual hemifield of presentation, entered as a fixed factor [model (2): GA ~ 1 | PNr + HA * hemifield].

Final models were calculated by removing nonsignificant interactions by backward elimination using the step function of the lmerTest package (Kuznetsova et al. 2017). Degrees of freedom were estimated using Satterthwaite's approximation, and p‐values rounded to the third decimal were considered significant if below 0.050.

## 3 Results

### 3.1 Time Series Decoding Identifies Global Precedence in Brain Activity

**FIGURE 2:**
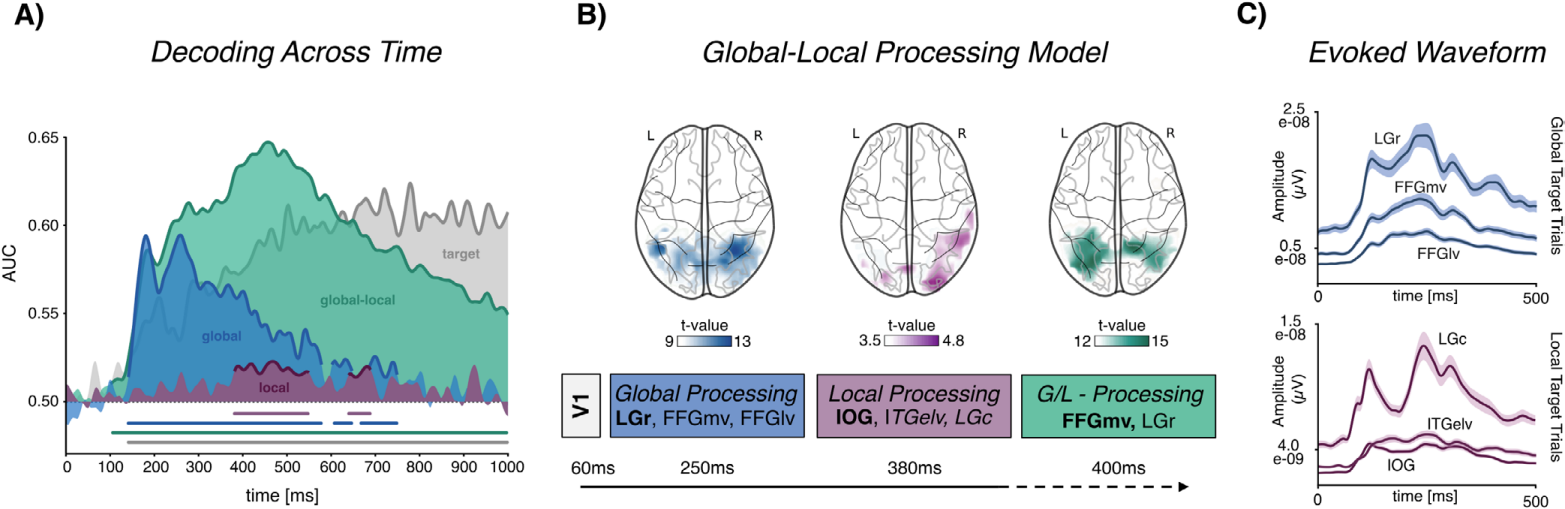
(A) Area under the curve (AUC) decoding accuracies across time for sensor‐level data including global, local, global–local and target contrasts. Bold lines indicate significant above 0.5 chance level AUC. (B) Global–Local Procesing Model: Information reaches V1 at ~60 ms. Global contrasts yield highest average AUCs at ~250 ms in LGr, FFGmv, mvOC l (see Table 2). Local contrasts yield highest average AUCs at ~380 ms in IOG, ITGelv and LGc. Global and local information may be integrated into comprehensive units and be most differentiable at ~400 ms in FFGmv and LGr. Source reconstruction plot is based on searchlight source level analysis results and span the top 25% of smoothed against chance level *t*‐values. (C) Absolute evoked waveforms for the three main global and local processing regions. c, caudal; FFGmv, Fusiform Gyrus medioventral; IOG, Inferior Occipital Gyrus; ITG, Inferior Temporal Gyrus; l, lateral; LG, Lingual Gyrus; mvOC, medioventral Occipital Cortex; r, rostral; V1, Primary Visual Cortex.

As a first step, we established a general time course of global–local processing by training and testing the classifier on sensor data for several stimulus contrasts (see Figure 1B). Contrasts included different visual hemifields, targets compared to non‐targets, different non‐target global letter forms for global processing (global contrast), different non‐target local letter forms for local processing (local contrast) as well as global targets compared to local targets (global–local contrast). To this end, we applied temporal decoding from 0 to 1000 ms after stimulus onset using linear discriminant analysis (LDA) with a fivefold cross‐validation and five repetitions with AUC used as a metric for decoding quality. The resulting time course is depicted in Figure 2A.

Visual hemifield (not shown) yielded robust and sustained above‐chance classification from 60 to 1000 ms (*p*_cluster_ < 0.001) and peak accuracy levels from 140 to 240 ms at ~96%. For target compared to non‐target related brain activity, AUC reached above chance levels from 140 to 1000 ms (*p*_cluster_ < 0.001) with sustained peak levels from 680 to 780 ms at ~63%. For the global contrast, AUC reached above chance levels from 140 to 580 ms (*p*_cluster_ < 0.001) and from 600 to 750 ms (*p*_cluster_ = 0.010) with peak accuracy levels from 170 to 290 ms at ~59%. For the local contrast, AUC reached above chance levels from 380 to 550 ms (*p*_cluster_ < 0.001) and from 640 to 690 ms with peak accuracy levels in both time windows at ~52.5%. For global compared to local target related brain activity, AUC reached above chance levels from 100 to 1000 ms (*p*_cluster_ < 0.001) with peak levels from 450 to 470 ms at ~65%, partly overlapping the local contrast peak time window. To provide an impression of signal quality in source space, absolute evoked waveforms from regions of interest (compare Section 3.2) are depicted in Figure 2C

Overall, time series decoding results provide the first MEG and MVPA evidence for global precedence, as global contrasts showed above‐chance and peak accuracy values ~210 ms before local contrasts. Also, while global–local target contrasts were decoded above chance as early as global contrasts, the most accurate distinction between both target levels is not made until each of its constituent levels, i.e., global and local letter forms, has both reached their individual decoding peaks.

### 3.2 Searchlight Analysis Confirms Previously Identified Core Regions

Peak accuracies of whole brain sensor‐level time series were used to define data‐driven time windows of interest for the subsequent whole‐brain searchlight analysis. Included areas contained time windows of 50–200 ms (first global peak) and 200–350 ms (second global peak) for the global contrast, as well as 350–500 ms for both the local contrast and the global compared to local target contrast. Searchlight analysis involved a fivefold cross‐validation LDA with 100 repetitions and included the three closest neighboring voxels.

All searchlight AUCs of the 2205 gray matter voxels were tested against chance using a Monte Carlo multiple‐comparison‐corrected cluster‐based permutation test with 10,000 iterations. Regions were included as ROIs if their average *t*‐value was at least 80% of the *t*‐value of the region with the maximum average *t*‐value (see Table 1).

**TABLE 1:** Mean *t*‐values of identified regions of interest from all contrasts based on comparisons of area under the curve (AUC) decoding accuracy against a 50% chance level.

ROI	Global	Global	Local	Global/local
	50–200 ms	200–350 ms	350–500 ms	350–500 ms
	*t*‐value	*t*‐value	*t*‐value	*t*‐value
CG v	4.31	9.61	1.22	12.45
CG d	3.44	9.18	1.36	10.43
FFG lv	5.33	10.40	2.66	12.83
FFG mv	6.00	10.91	3.18	13.76
IOG	5.81	10.21	3.69	11.89
IPL c	5.57	9.05	2.27	11.73
IPL rv	4.99	9.28	1.75	11.12
ITG cl	3.13	8.47	2.20	11.17
ITG elv	5.11	9.76	3.23	12.42
ITG vl	5.24	9.69	2.67	12.37
LG c	6.25	10.24	3.44	10.24
LG r	5.80	10.99	2.36	13.21
V5	6.16	10.24	3.21	12.73
MOG	6.12	9.98	3.08	12.00
MTG dl	4.62	9.51	2.20	11.98
MVOC c	5.45	9.23	2.60	9.84
MVOC r	5.86	9.84	2.30	11.04
MVOC vmpos	5.66	10.21	1.43	12.58
OPC	4.79	8.70	3.11	9.44
PC (A31)	3.95	9.84	1.40	10.50
PHG TH	3.67	9.11	2.02	12.02
PC dmpos	5.01	9.90	1.01	12.48
SOG l	4.66	8.77	1.31	11.07
SOG m	4.37	8.91	1.95	10.59
STS cp	4.39	9.20	2.23	10.80

*Note:* Double‐indented values represent regions with the maximum average *t*‐values for each contrast. Indented values correspond to regions with a mean *t*‐value ≥ 80% of the maximum region *t*‐value and were included for asymmetry profile computation. N‐trials global, local = 300; N‐trials global–local = 600.

Abbreviations: c, caudal; CG, Cingulate Gyrus; d, dorsal; e, extreme; FFG, Fusiform Gyrus; IOG, Inferior Occipital Gyrus; IPL, Inferior Parietal Lobule; ITG, Inferior Temporal Gyrus; l, lateral; LG, Lingual Gyrus; LOC, Later Occipital Cortex; m, medial; MOG, Medial Occipital Cortex; MTG, Middle Temporal Gyrus; MVOC, Medioventral Occipital Cortex; OPC, Occipital Polar Cortex; p, posterior; PC, Precuneus; PHG, Parahippocampal Gyrus; POS, Parietooccipital Sulcus; r, rostral; SOG, Superior Occipital Gyrus; STS, Superior Temporal Sulcus; v, ventral; vmpos, ventromedial parietooccipital.

For the global contrast, regions with the highest against chance level *t*‐values shift from earlier visual processing areas like caudal Lingual Gyrus (LGc) and Lateral Occipital Cortex (V5) during 50–200 ms to higher‐order sections of the ventral visual stream, such as rostral Lingual Gyrus (LGr), medioventral Fusiform Gyrus (FFGmv), and lateroventral Fusiform Gyrus (FFGlv) during 200–350 ms (see Table 1). For the local contrast (350–500 ms), regions with the highest against chance level *t* values were situated around earlier visual areas including the Inferior Occipital Gyrus (IOG), extreme lateroventral Inferior Temporal Gyrus (ITGelv) and LGc (see Table 1). For the global versus local target contrast (350–500 ms), the highest against chance *t* values were located around higher‐order sections of the visual system, including FFGmv and FFGlv, as well as LGr (see Table 1).

### 3.3 Searchlight Asymmetries Question Conventional Assumptions About Hemispheric Lateralization

Lateralization indices (LI) for each contrast were calculated across the entire sample (Table 2). Indices were calculated between (1) whole hemispheres, (2) literature‐based ROIs, and (3) data‐driven ROIs. While global processing only indicates lateralization for whole hemisphere comparisons, local contrast LI exhibits right‐hemispheric lateralization of processing throughout all measures.

**TABLE 2:** Lateralization indices (LI) for the global and local processing contrast, for whole hemisphere comparisons, literature‐based ROIs, and data‐driven ROIs.

Comparison	Lateralization index (LI)	*p*	bF
Global contrast			
Whole hemisphere	−0.01	0.003	3.43
Literature ROIs (LG)	0.14	0.189	0.65
Data‐driven ROIs	−0.07	0.273	0.18
Local contrast			
Whole hemisphere	0.19	< 0.001	2.46E+10
Literature ROIs (OccInf)	0.53	0.011	4.98
Data‐driven ROIs	0.20	< 0.001	1.24E+3

*Note:* Negative indices indicate a left‐hemispheric advantage. p‐values are derived from voxel‐based permutation tests. bF represents Bayes factors for voxel‐based hemispheric comparisons.

Abbreviations: LG, Lingual Gyri: LGr, LGc; OccInf, Inferior Occipital Gyrus.

**FIGURE 3:**
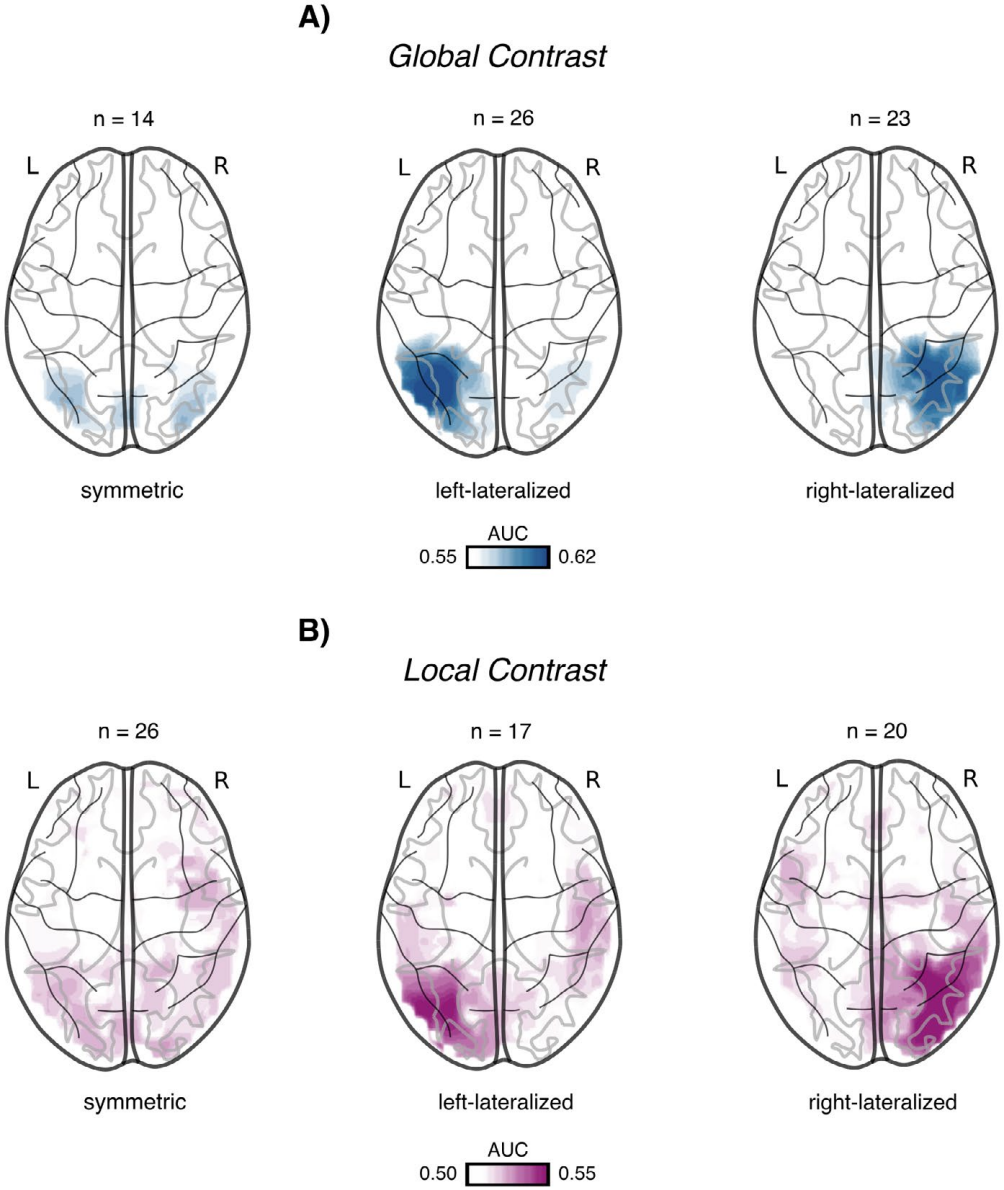
Lateralization profiles. (A) Global contrast source projections of whole‐brain area under the curve (AUC) searchlight decoding accuracies for the symmetric (*n* = 14), left‐dominant (*n* = 26), and right‐dominant (*n* = 23) lateralization groups. (B) Local contrast source projections of whole‐brain searchlight decoding accuracies for the symmetric (*n* = 26), left‐dominant (*n* = 17), and right‐dominant (*n* = 20) lateralization groups.

After a thorough assessment of each subject's searchlight results, we discovered substantial variability in asymmetry patterns across our sample. In order to quantify the encountered heterogeneity in lateralization for each contrast, within‐subject Bayesian t‐tests were applied for hemispheric comparison of ROI AUCs, resulting in three distinct lateralization profiles per contrast *global*: symmetric (*n* = 14, all Bayes factors (bf) < 0.97), left (*n* = 26, all bf > 5.43), right (*n* = 23, all bf > 5.53); *local*: symmetric (*n* = 26, all bf < 4.98), left (*n* = 17, all bf > 6.86), right (*n* = 20, all bf > 12.90) (Figure 3, see Supporting Information for individual values).

To assess the internal consistency of the discovered lateralization profiles for global and local processing contrasts, we computed the difference between left and right hemispheric decoding accuracies for each of the 100 repetition result matrices of our fivefold cross‐validations and assessed the resulting distribution of hemispheric lateralization with a two‐tailed binomial test *global*: symmetric (mean percentage right hemispheric dominance (M_RH_) = 52.50%, range = 12%–81%), left (M_RH_ = 2.46%, range = 0%–17%), right (M_RH_ = 98.65%, range = 88%–100%); *local*: symmetric (M_RH_ = 48%, range = 15%–81%), left (M_RH_ = 3.88%, range = 0%–21%), right (M_RH_ = 96.05%, range = 83%–100%; see Table S1A,B for individual values). Since each repetition uses a different subset of trials for training and testing, this method enables us to assess the reliability of the discovered lateralization profiles while avoiding significant classifier performance drops due to insufficient trial numbers.

Moreover, in order to assess the general consistency across different training‐ and test‐trial subsets, we computed an Interclass Correlation Coefficient (ICC) among all 100x2205x1 result vectors for each subject. Global contrast ICC_Fisher_ averaged 0.84, ranging from good to excellent (0.73–0.94), whereas local contrast ICC_Fisher_ averaged at 0.73, ranging from moderate to good (0.53–0.86) (see Table S1A,B for individual values).

Altogether, whole‐brain searchlight analysis identified previously established brain areas for global–local processing (Fink et al. 1996; Fink, Halligan, et al. 1997; Han et al. 2002), with statistically most significant decoding accuracies within LG for global level processing and IOG for local level processing. However, inspection of individual searchlight AUCs revealed a striking heterogeneity in lateralization patterns across the study sample, challenging the idea of a universal global LVF–RH and local RVF–LH asymmetry for localized AUC decoding accuracies. To further explore hemispheric AUC asymmetries in global–local processing, irrespective of lateralization, we averaged absolute difference scores between ROIs of each hemisphere across the asymmetric sample and compared these averages against zero (see Supporting Information for detailed values). For both the global and the local contrast, LG demonstrated significantly lower hemispheric asymmetries than IOG (Figure S1). Additionally, while both contrasts showed constant significant asymmetries throughout each stimulus trial, local contrast asymmetries remained constant, whereas global contrast asymmetries followed the global AUC envelope (Figure S2).

### 3.4 Local Contrast Processing Accuracies Related to Behavioral Global Advantage Effects

To test whether reactions to global targets were significantly faster than reactions to local targets, we calculated the global advantage effect as a standardized contrast (Zhang 2010), i.e., the difference in mean reaction times between local and global targets divided by the standard deviation, for each participant before entering group analysis. One‐sample t‐tests were performed comparing GA to zero. Reactions to global targets were significantly faster than reactions to local targets (*t*(61) = 9.39, *p* < 0.001, *d* = 1.19). Within our sample, 56 subjects showed the expected positive GA, while 6 subjects showed a negative GA, that is, faster reactions to local than global target letters. The split‐half Spearman‐brown‐corrected correlation showed a moderate reliability of global precedence for both GA (rho = 0.70, *p* < 0.001) and absolute reaction time differences (rho = 0.66, *p* < 0.001). There were no significant differences in GA between visual hemifield presentation conditions (*b* = −0.05, SEb = 0.08, *t*(122) = −0.43, *p* = 0.555).

**FIGURE 4:**
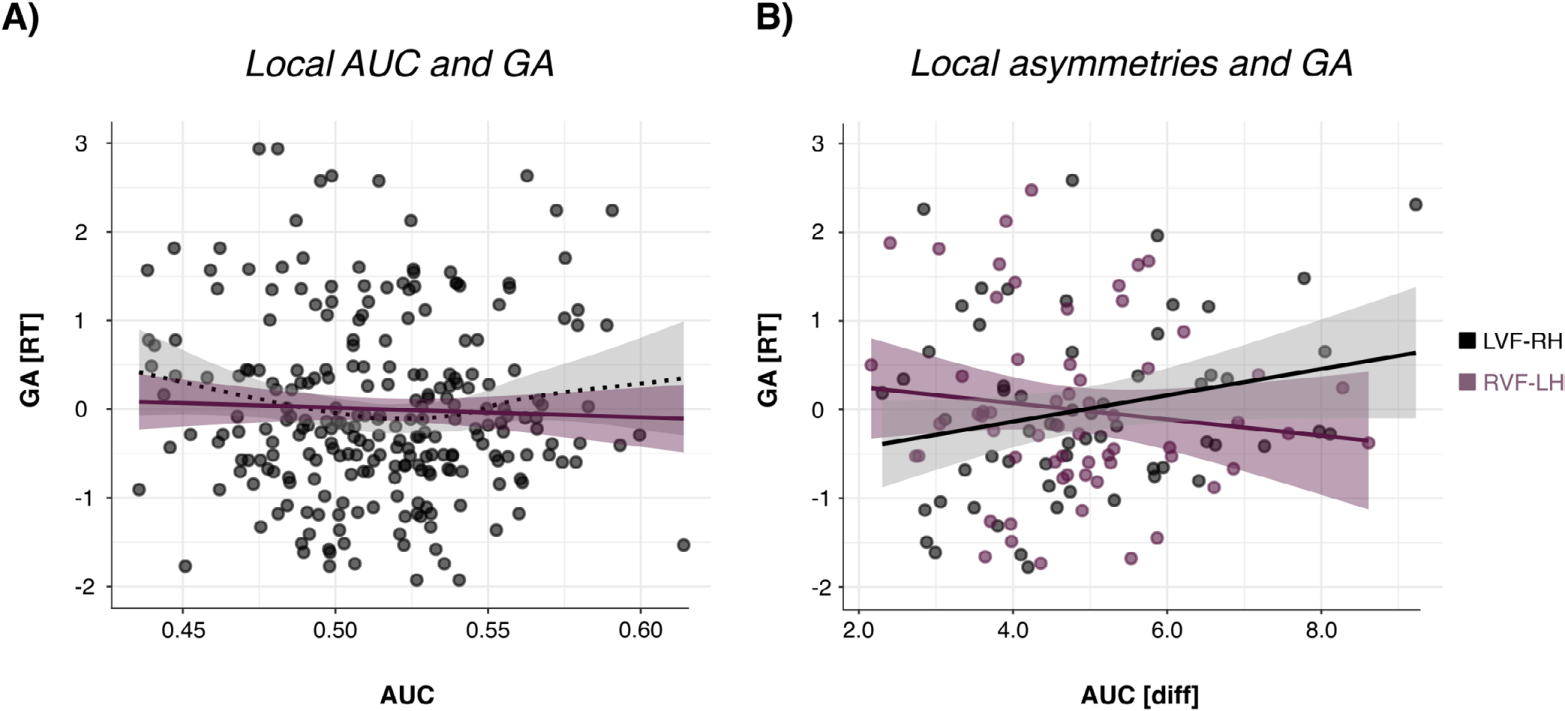
(A) Relationship between local contrast area under the curve (AUC) decoding accuracies and the global advantage effect (GA). (B) Interaction between hemifield of presentation and hemispheric asymmetry (absolute AUC difference between hemispheres) on GA. GA is positively related to hemispheric asymmetries for initial left visual field—right hemisphere (LVF—RH) presentation of local information but negatively related to hemispheric asymmetries for initial RVF—LH presentati.

Regarding the relationship of GA to decoding accuracies, our analysis revealed a significant main effect of local contrast AUC on GA (*b* = −3.80, SEb = 1.24, t(206.76) = −3.07, *p* = 0.002), indicating that GA decreases as local contrast decoding accuracies increase (Figure 4A). On the other hand, global contrast AUC did not show a significant effect on GA (b = 1.28, SEb = 1.04, *t*(223.16) = 1.23, *p* = 0.220).

To assess the impact of hemispheric AUC asymmetries on behavioral measures, the absolute difference in decoding accuracies between each left and right‐hemispheric ROI was calculated and used as a measure of hemispheric advantage (HA). For both the global and local decoding contrast, there was a significant interaction of HA and visual field on GA (global: *b* = 3.38, SEb = 0.51, *t*(2664.88) = 6.61, *p* < 0.001; local: *b* = −3.14, SEb = 1.17, t(806.75) = −2.67, *p* = 0.008). For the global contrast, HA was negatively related to GA with both RVF‐LH presentation (*b* = −5.21, SEb = 8.87, *t*(60) = −0.59, *p* = 0.559) and LVF‐RH presentation (*b* = −8.34, SEb = 6.82, *t*(60) = −1.22, *p* = 0.227) (Figure 4B). For the local contrast, HA was negatively related to GA with RVF‐LH presentation (*b* = −9.15, SEb = 9.60, *t*(60) = −0.95, *p* = 0.345), but positively related to GA with LVF‐RH presentation (*b* = 14.84, SEb = 8.08, *t*(60) = 1.84, *p* = 0.071). Thus, global precedence seems to be generally impaired by the lateralization of information processing (i.e., higher HA), but not by the lateralization of local processing after RVF‐LH presentation. When examining this relationship for different local processing lateralization profiles, the positive relationship between GA and HA was only indicated for the right‐lateralized group (*b* = 16.86, SEb = 9.91, *t*(38) = 1.70, *p* = 0.097) but not for the left‐lateralized group (*b* = −1.37, SEb = 13.56, *t*(30) = 1.70, *p* = 0.920).

Overall, we observed a negative relationship between local letter contrast decoding accuracies and GA, suggesting a dominant role of local‐level processing in GA variability. Additionally, lateralization of global and local processing (as indicated by AUC) seems to inhibit GA, except for lateralization of local processing after LVF‐RH presentation, which was related to increased GA.

## 4 Discussion

In an attempt to dissect the temporal and spatial dynamics of global–local processing, we applied MVPA on MEG recordings from 63 participants performing a divided attention divided visual field Navon paradigm. Using various stimulus contrasts (Figure 1B), we mapped critical time periods and brain regions involved in different processing stages, starting with early lateralization, progressing to lower‐level feature identification, and culminating in global–local integration. To achieve this, we aimed to identify regions of interest that, according to MVPA, contribute the most informative activity to the discrimination of stimulus aspects during contrast‐specific time windows of interest. Regions with increased informative activity yielded greater differences in multivariate activity and are thus considered specialized key nodes for processing the respective stimulus aspects, such as global or local letter shape. Furthermore, we investigated variations in hemispheric lateralization for global–local processing across individuals as well as their relation to behavioral performance.

### 4.1 Temporal Dynamics

Sensor‐level MVPA of whole‐brain activity revealed distinct time windows of interest for all five of the examined contrasts (Figure 2A). As expected, the visual hemifield was the first condition displaying significant above‐chance‐level decoding as early as 60 ms after stimulus onset, aligning with prior MEG experiments establishing a 50–80 ms time window for the arrival of visual information in V1 (Vanni et al. 2001; Vanni et al. 2004).

Next, global stimulus letter contrasts reached significant above‐chance accuracies around 140 ms, peaking from 170 to 290 ms at ~59%, with distinct spikes at ~160 ms and ~250 ms, respectively. The first spike for global letter decoding matches ERP evidence of visual stimulus categorization occurring as early as 150 ms after stimulus onset (De Cesarei et al. 2015; Fabre‐Thorpe et al. 2001). Also, ERP findings on the time course of letter processing have suggested a lower level visual similarity effect in letter recognition between 120 ms and 220 ms, followed by a more abstract effect between 220 ms and 300 ms (Petit et al. 2006). Similarly, the two distinct global processing spikes in the current study may represent differentiability based on mere visual similarity (~160 ms) and letter categorization (~250 ms). This distinction is particularly interesting when considering the progression in the visual processing hierarchy observed for each spike, shifting from the ventral visual stream's posterior to more object‐specific anterior regions.

Only at a later stage, contrasts of different local stimulus letters reached significant above‐chance decoding accuracies between 380 ms and 550 ms with a comparatively smaller peak of ~52.5% around 470 ms. The observed time delay of ~150 ms between hierarchical levels provides the first MEG and MVPA‐based evidence for global precedence. This finding aligns well with ERP sequences from top‐down facilitated selective attention paradigms, displaying comparatively higher N1 (150–200 ms) components for global attention and relatively higher N2 components (250–300 ms) for local attention (Evans et al. 2000). Since the stimulus material of the current study did not allow for a comparison of two different local letters in the absence of a global target, reduced local contrast decoding accuracies may be attributable to global precedence effects. While local non‐targets may not have received sufficient processing to elicit a clearly discernible brain response, global non‐targets were still highly decodable as they naturally precede in the processing chronology. Yet, target‐based contrasts might have given an even better insight into local processing time courses and localization. These findings on global precedence are also mirrored by behavioral response time measures, as responses to global targets were significantly faster compared to responses to local targets, as indicated by the global advantage effect (GA). More specifically, our findings indicate a significant negative relationship between GA and local contrast decoding accuracies, suggesting a central role of local letter processing in the emergence of GA.

Finally, global–local contrasts were decoded above chance as early as 80 ms, with peak accuracies of ~64% immediately following and partly overlapping with local contrast maxima from 400 to 500 ms. Thus, although global targets should already be discriminable from local targets upon V1 arrival, the strongest distinction between both levels is made as soon as they have reached their individual decoding peaks.

### 4.2 Source Localization of Global–Local Processing

In line with pioneering PET studies (Fink et al. 1996; Fink, Halligan, et al. 1997), we located the statistically most significant global contrast decoding accuracies within the LG, followed by other previously reported occipito‐temporal areas (Fink et al. 1996; Fink, Halligan, et al. 1997; Robertson and Lamb 1991; Robertson et al. 1988) (see Table 1 for details). Between the two identified spikes (~160 ms and ~250 ms), AUC hotspots shifted from more basal, posterior visuospatial processing areas to anterior, object‐specific locations.

For the local contrast processing peak (~350 ms), we again matched earlier results (Fink et al. 1996; Fink, Halligan, et al. 1997; Han et al. 2002), as the most significant decoding accuracies were located within the IOG, followed by other previously reported areas (see Table 1 for details). In line with an increasing receptive field size along the ventral visual stream (Guclu and van Gerven 2015; Kobatake and Tanaka 1994), fMRI‐based decoding studies on face processing have previously identified a continuum from ventral posterior‐configural to ventral anterior‐holistic spatial processing regions (de Haas et al. 2016; de Haas et al. 2021). Likewise, our results suggest that this continuum likely extends to other complex objects such as hierarchical letters, with the IOG and the more anterior LG as key players.

Finally, AUC peaks for contrasting local and global target letters (~450 ms) provided the most statistically significant decoding accuracies within the FFG, followed by previously identified regions involved in processing both hierarchical levels. The FFG is involved in the integration of visual features into cohesive perceptual wholes (Gerlach et al. 2002) and encompasses higher‐level visual processing areas, such as the face, shape, or visual word form areas, close to our current AUC peak region. The special role of the FFG in global–local contrast decoding may therefore partly indicate the integration of global and local stimulus aspects into a superordinary percept and represent a relation similar to that of whole words to their constituent letters.

### 4.3 Hemispheric Asymmetries in Global–Local Processing

Challenging the common assumption of right‐hemispheric specialization for global processing and left‐hemispheric specialization for local processing, lateralization indices (LI) indicated a significant right‐hemispheric lateralization of local processing. A closer examination of individual searchlight results revealed three distinct lateralization profiles across our sample for the global and local contrast, respectively: symmetric, left‐hemispheric dominance, and right‐hemispheric dominance (Figure 3). These profiles were fairly evenly distributed, with individuals displaying lateralization for none, one, or both hierarchical levels, either within the same or opposite hemispheres.

Correspondingly, our behavioral results did not confirm the assumed global‐right local‐left processing advantage, as there was no significant effect of the hemifield of presentation on GA. Furthermore, GA was generally reduced with increased hemispheric lateralization but benefited from increased local contrast decoding lateralization when information was presented in the left visual field. Although this aligns with the traditional view of the right hemisphere lacking specialization for local‐level processing, the heterogeneous lateralization profiles discovered in our sample made this result somewhat unexpected. However, when analyzing each of the encountered lateralization profiles separately, the relationship between GA and lateralization was only positive for individuals with the corresponding right hemispheric lateralization of local processing.

The divergence between the observed lateralization of global–local processing in this study and a majority of previous research may stem from a variety of factors, reflecting both interindividual and methodological differences. First, there might be a significant influence of sampling effects. Interindividual variance in visuospatial lateralization has already been reported for other study samples (Banich et al. 1992; Gevins and Smith 2000; O'Boyle et al. 1991; Wendt and Risberg 1994). Such differences may arise, for instance, from variations in cognitive processing styles that could be influenced by an individual's degree of verbal or spatial competence and the corresponding demands imposed by a given stimulus (Gevins and Smith 2000). Individual variance in processing style and subsequent laterality may therefore represent a crucial but commonly overlooked factor that may have prevented a considerable number of earlier studies (Alivisatos and Wilding 1982; Boles and Karner 1996; Hubner 1997; Martin 1979; Mena 1992; Polich and Aguilar 1990; Van Kleeck 1989) from identifying the regular global‐right, local‐left asymmetry pattern.

Second, methodological differences may explain the observed lateralization differences. Previous imaging studies using PET or fMRI (Fink et al. 1996; Fink, Halligan, et al. 1997; Fink, Marshall, et al. 1997; Han et al. 2002) offer high spatial resolution but rely on hemodynamic signals, reflecting changes in blood flow as an indirect marker of neural activity. Due to their low temporal resolution, these signals reflect hemodynamic changes that integrate neural activity over seconds, effectively averaging responses across entire trials or blocks. In contrast, EEG and MEG capture rapid fluctuations in electromagnetic fields generated by synchronized neural activity, providing fine‐grained temporal resolution at the scale of milliseconds (Liu and He 2008). However, since previous EEG studies typically assessed hemispheric differences by comparing left and right electrode activity, even simple changes in dipole orientation may have produced apparent asymmetries at the scalp level without actual asymmetries in source locations. The current study mitigates this issue and provides a more accurate spatial representation of the involved neural dynamics by employing source reconstruction and leveraging MEG's insensitivity to the anisotropic conductivity of brain tissue. Moreover, the present MEG study applied multivariate searchlight decoding in precise 150 ms windows with carefully designed contrasts to minimize irrelevant stimulus differences. This approach captures distinct stages of global–local differentiation while reducing confounds from higher‐order processes such as attention switching, conflict resolution, or decision‐making. However, while providing a more granular perspective, this also filters out broader mechanisms that are integral to the task. Thus, studies capturing multiple processes at once remain just as important, and future use of complementary methods such as MVPA may provide additional insights also into these other processes. Moreover, since previous univariate analyses assessed activity voxel by voxel or sensor by sensor, hemispheres with stronger mean activation in response to a given stimulus were identified as dominant (Coutanche 2013). In contrast, searchlight analysis detects subtle neural differences in contrast‐specific multivariate activation patterns, potentially attributing dominance even to regions with lower mean activation.

Overall, these distinctions suggest that the current findings do not necessarily contradict prior evidence on global–local lateralization, but rather offer a complementary perspective. While PET and fMRI studies have shown hemispheric dominance for global and local processing based on broad and overlapping neural processes, MEG's spatial and temporal precision allows for the examination of specific neural representations at different processing stages. This may reveal subtle, localized effects and individual variability in lateralization that univariate methods might miss. However, univariate analyses of electrophysiological data have also shown deviations from the typical global‐right/local‐left lateralization pattern (Han et al. 1997, 1999) and laterality in the current study was not only divergent from previous results but also heterogeneous across individuals. Thus, we cannot rule out a potential impact of sampling effects on the observed laterality results.

### 4.4 Conclusions

The combination of MEG and MVPA offered specific insights into different steps of global–local processing. Our results outline a potential model of global–local processing with unique temporal and spatial precision (Figure 2B) that may serve as a starting point for future studies to build on and extend through additional task and stimulus manipulations. By employing across‐time decoding and searchlight source analysis, this study is, to our knowledge, the first to demonstrate MEG‐ and MVPA‐based evidence for global precedence, confirming previously separate findings on hierarchical letter processing from time or source domains within a single dataset. Unlike a majority of earlier investigations, our MVPA results could not confirm the commonly accepted global‐right local‐left asymmetry pattern in hierarchical letter processing, as lateralization profiles displayed high interindividual variation. While methodological differences may have contributed to this divergence, our results introduce interindividual variability in laterality as a potential factor in past inconsistencies regarding hemispheric dominance and advocate for considering such differences in future asymmetry research. As laterality has previously been shown to rest upon factors such as sex or age (Clements et al. 2006; Yu et al. 2014), as well as the interplay of task properties and individual cognitive proficiency, subsequent studies should investigate how demographic, developmental, and educational variables contribute to the observed diversity in lateralization patterns.

## Author Contributions

**Tobias Hausinger:** data curation, formal analysis, methodology, visualization, writing – original draft. **Patrick Reisinger:** formal analysis, visualization, writing – review and editing. **Nathan Weisz:** methodology, writing – review and editing. **Andrea Hansen:** data curation, formal analysis, investigation. **Ti‐Anni Harris:** formal analysis, investigation. **Belinda Pletzer:** conceptualization, data curation, formal analysis, funding acquisition, investigation, methodology, project administration, supervision, writing – review and editing.

## Conflicts of Interest

The authors declare no conflicts of interest.

## Supporting information


Data S1.

## Data Availability

Data and code necessary for statistical analysis and generating figures are available at (https://osf.io/fs8t5/?view_only=e50cd9ef28734e32a13b49907d17a3w3b) (Hausinger et al. 2024). Raw data are available from the corresponding author upon reasonable request.
