# Supplementary material for: Spatio‐Temporal Decoding of the Navon Task Challenges Rigid Hemispheric Asymmetries in Global–Local Processing
Source: Psychophysiology. 2025 Mar 4;62(3):e70032. doi: 10.1111/psyp.70032 (PMC11879918; doi:10.1111/psyp.70032)
Supplement: Supplementary file 1 — Data S1. [file PSYP-62-e70032-s001.docx]

Supplementary Figures:


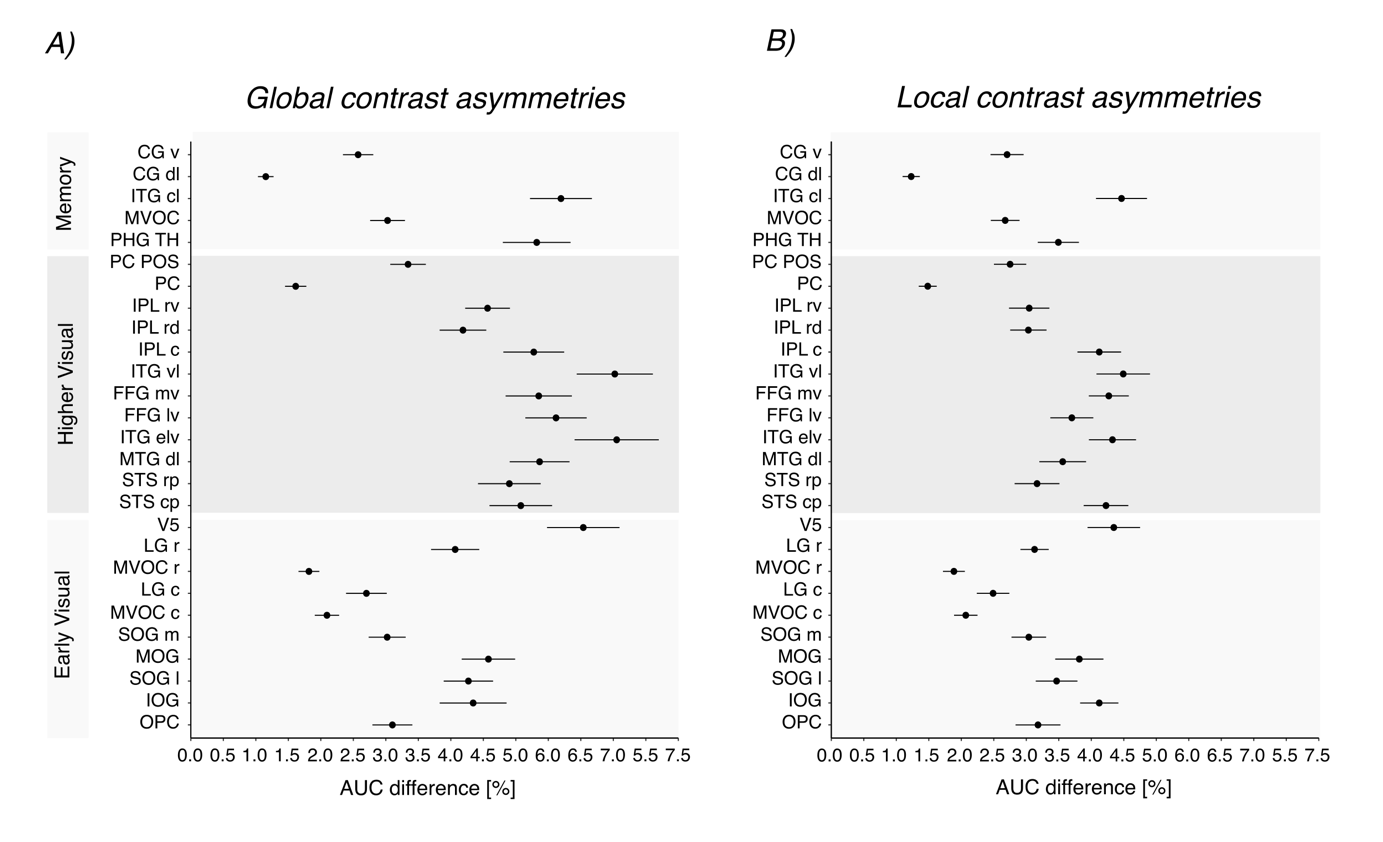


**Supplementary Figure 1**: Absolute area under curve difference (AUC difference) in searchlight decoding accuracies between hemispheres in early visual, higher visual and memory related areas for the global **A)** and local **B)** level contrast. While as commonly expected (Moscovitch, Scullion, & Christie, 1976), higher visual processing areas generally showed a higher degree of hemispheric decoding asymmetries, the typically more object specific LG showed a lower degree of hemispheric decoding asymmetries for both the global and local contrast. This divergence from the expected pattern is notable, as visual processing areas like the LG are typically associated with higher hemispheric specialization compared to basic regions such as IOG and middle occipital gyrus (MOG). Consequently, one would anticipate greater lateralization in neural activity or decoding accuracies for these regions.


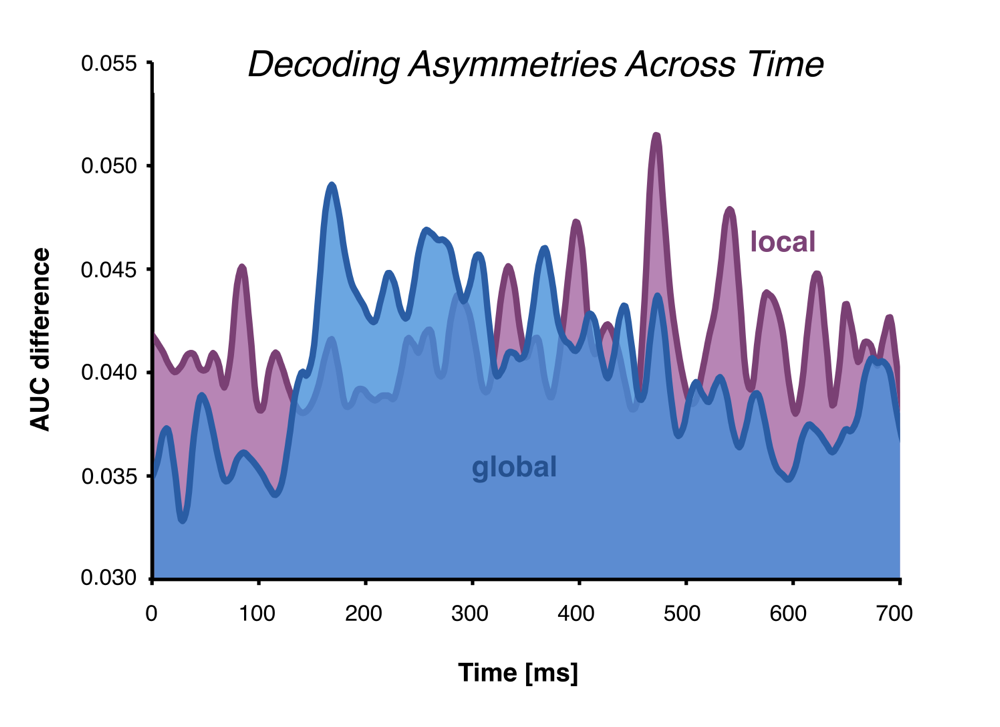


**Supplementary Figure 2:** Time series of absolute area under the curve decoding accuracy differences (AUC diff) between ROIs of each hemisphere for the global and local level contrast. Comparisons against zero were significant throughout displayed time-window (p_cluster_ < .001 for both contrasts).

Supplementary Tables:

1. **Contrast Lateralization**

*bf (Bayes Factor, comparison between hemispheres), subj (Subject Number), lh/rh (Searchlight Decoding Accuracies left/right Hemisphere), lat (Lateralization)*

1. *Global Contrast (200-350ms)*

| bf | subj | lh | rh | lat | % rh | p % | ICC |
| --- | --- | --- | --- | --- | --- | --- | --- |
| 8.30E-01 | 1 | 0.61 | 0.60 | sym | 12 | < .001 | 0.89 |
| 3.71E+08 | 3 | 0.55 | 0.53 | left | 1 | < .001 | 0.79 |
| 2.31E+09 | 4 | 0.54 | 0.57 | right | 100 | < .001 | 0.86 |
| 1.66E+03 | 5 | 0.54 | 0.56 | right | 97 | < .001 | 0.85 |
| 1.44E+15 | 7 | 0.60 | 0.57 | left | 0 | < .001 | 0.78 |
| 1.93E-01 | 8 | 0.60 | 0.60 | sym | 37 | .012 | 0.84 |
| 3.78E+09 | 9 | 0.62 | 0.59 | left | 0 | < .001 | 0.84 |
| 1.23E-01 | 12 | 0.57 | 0.57 | sym | 57 | .193 | 0.71 |
| 5.44E+00 | 15 | 0.67 | 0.66 | left | 4 | < .001 | 0.91 |
| 2.08E+22 | 16 | 0.58 | 0.53 | left | 0 | < .001 | 0.86 |
| 3.37E+04 | 18 | 0.55 | 0.53 | left | 7 | < .001 | 0.71 |
| 2.65E-01 | 20 | 0.57 | 0.56 | sym | 25 | < .001 | 0.85 |
| 1.25E+32 | 22 | 0.65 | 0.59 | left | 0 | < .001 | 0.91 |
| 9.71E-01 | 23 | 0.54 | 0.55 | sym | 81 | < .001 | 0.86 |
| 1.83E+04 | 25 | 0.56 | 0.58 | right | 97 | < .001 | 0.81 |
| 9.39E+02 | 27 | 0.53 | 0.55 | right | 94 | < .001 | 0.79 |
| 2.16E+18 | 30 | 0.56 | 0.60 | right | 100 | < .001 | 0.90 |
| 1.00E+18 | 31 | 0.59 | 0.63 | right | 100 | < .001 | 0.85 |
| 1.19E+43 | 32 | 0.64 | 0.58 | left | 0 | < .001 | 0.90 |
| 1.98E-01 | 33 | 0.55 | 0.56 | sym | 69 | < .001 | 0.87 |
| 8.41E+10 | 38 | 0.57 | 0.53 | left | 0 | < .001 | 0.87 |
| 1.18E+33 | 45 | 0.52 | 0.55 | right | 100 | < .001 | 0.75 |
| 5.54E+00 | 46 | 0.58 | 0.59 | right | 88 | < .001 | 0.85 |
| 2.83E+17 | 48 | 0.50 | 0.53 | right | 100 | < .001 | 0.78 |
| 1.32E+06 | 49 | 0.59 | 0.61 | right | 100 | < .001 | 0.88 |
| 3.66E+25 | 51 | 0.53 | 0.57 | right | 100 | < .001 | 0.82 |
| 6.42E+22 | 53 | 0.60 | 0.56 | left | 0 | < .001 | 0.82 |
| 1.30E-01 | 54 | 0.55 | 0.55 | sym | 60 | .057 | 0.79 |
| 1.02E-01 | 55 | 0.54 | 0.54 | sym | 52 | .764 | 0.67 |
| 9.32E+18 | 61 | 0.52 | 0.49 | left | 0 | < .001 | 0.77 |
| 1.64E-01 | 63 | 0.51 | 0.51 | sym | 65 | .004 | 0.79 |
| 2.10E+04 | 65 | 0.62 | 0.60 | left | 2 | < .001 | 0.87 |
| 6.27E+02 | 68 | 0.54 | 0.53 | left | 9 | < .001 | 0.77 |
| 1.64E+05 | 72 | 0.62 | 0.64 | right | 99 | < .001 | 0.88 |
| 2.85E-01 | 75 | 0.56 | 0.56 | sym | 70 | < .001 | 0.76 |
| 8.21E+17 | 84 | 0.60 | 0.65 | right | 100 | < .001 | 0.92 |
| 1.50E+28 | 93 | 0.57 | 0.61 | right | 100 | < .001 | 0.85 |
| 3.75E+06 | 97 | 0.66 | 0.63 | left | 0 | < .001 | 0.92 |
| 1.49E+18 | 107 | 0.60 | 0.63 | right | 100 | < .001 | 0.86 |
| 9.61E+02 | 111 | 0.62 | 0.60 | left | 1 | < .001 | 0.90 |
| 1.22E+52 | 113 | 0.52 | 0.62 | right | 100 | < .001 | 0.94 |
| 3.47E+11 | 116 | 0.54 | 0.57 | right | 100 | < .001 | 0.79 |
| 3.12E+14 | 121 | 0.55 | 0.51 | left | 0 | < .001 | 0.85 |
| 2.84E+05 | 123 | 0.55 | 0.53 | left | 1 | < .001 | 0.81 |
| 2.29E+06 | 125 | 0.58 | 0.57 | left | 9 | < .001 | 0.70 |
| 3.55E+13 | 127 | 0.55 | 0.58 | right | 100 | < .001 | 0.84 |
| 1.78E+03 | 129 | 0.56 | 0.54 | left | 1 | < .001 | 0.80 |
| 1.46E+03 | 131 | 0.61 | 0.59 | left | 3 | < .001 | 0.88 |
| 4.99E+01 | 132 | 0.57 | 0.56 | left | 17 | < .001 | 0.79 |
| 1.35E+48 | 134 | 0.53 | 0.48 | left | 0 | < .001 | 0.78 |
| 9.35E+06 | 135 | 0.58 | 0.56 | left | 0 | < .001 | 0.82 |
| 4.12E+11 | 138 | 0.57 | 0.53 | left | 0 | < .001 | 0.85 |
| 5.60E+19 | 139 | 0.56 | 0.61 | right | 100 | < .001 | 0.91 |
| 5.24E+04 | 142 | 0.55 | 0.53 | left | 9 | < .001 | 0.78 |
| 1.95E-01 | 143 | 0.54 | 0.53 | sym | 32 | < .001 | 0.74 |
| 4.49E-01 | 144 | 0.59 | 0.60 | sym | 74 | < .001 | 0.82 |
| 1.00E+10 | 146 | 0.55 | 0.58 | right | 100 | < .001 | 0.85 |
| 2.80E+15 | 147 | 0.52 | 0.56 | right | 100 | < .001 | 0.85 |
| 1.35E+24 | 150 | 0.61 | 0.58 | left | 0 | < .001 | 0.81 |
| 8.53E-01 | 151 | 0.52 | 0.53 | sym | 73 | < .001 | 0.78 |
| 2.18E+04 | 153 | 0.60 | 0.62 | right | 94 | < .001 | 0.80 |
| 3.62E-01 | 154 | 0.57 | 0.57 | sym | 28 | < .001 | 0.82 |
| 4.38E+17 | 200 | 0.50 | 0.53 | right | 100 | < .001 | 0.72 |

1. *Local Contrast (350-500ms)*

| bf | subj | lh | rh | lat | % rh | p % | | ICC | |
| --- | --- | --- | --- | --- | --- | --- | --- | --- | --- |
| 3.64E+02 | 1 | 0.54 | 0.51 | left | 2 | < .001 | 0.76 | |  |
| 3.80E+23 | 3 | 0.49 | 0.56 | right | 100 | < .001 | 0.82 | |  |
| 1.76E-01 | 4 | 0.53 | 0.54 | sym | 51 | .920 | 0.78 | |  |
| 5.81E+09 | 5 | 0.51 | 0.56 | right | 100 | < .001 | 0.82 | |  |
| 4.64E+13 | 7 | 0.49 | 0.53 | right | 100 | < .001 | 0.75 | |  |
| 3.19E-01 | 8 | 0.55 | 0.54 | sym | 27 | < .001 | 0.63 | |  |
| 2.87E-01 | 9 | 0.51 | 0.50 | sym | 34 | .002 | 0.72 | |  |
| 3.07E+00 | 12 | 0.52 | 0.53 | sym | 76 | < .001 | 0.74 | |  |
| 1.29E+01 | 15 | 0.54 | 0.56 | right | 87 | < .001 | 0.76 | |  |
| 2.60E-01 | 16 | 0.52 | 0.52 | sym | 63 | .012 | 0.62 | |  |
| 1.92E-01 | 18 | 0.49 | 0.49 | sym | 39 | .035 | 0.70 | |  |
| 1.22E+00 | 20 | 0.52 | 0.51 | sym | 24 | < .001 | 0.62 | |  |
| 1.75E+00 | 22 | 0.53 | 0.52 | sym | 20 | < .001 | 0.68 | |  |
| 7.27E+01 | 23 | 0.53 | 0.55 | right | 91 | < .001 | 0.72 | |  |
| 8.98E+16 | 25 | 0.55 | 0.50 | left | 0 | < .001 | 0.74 | |  |
| 6.86E+00 | 27 | 0.47 | 0.45 | left | 21 | < .001 | 0.66 | |  |
| 1.12E+06 | 30 | 0.49 | 0.52 | right | 97 | < .001 | 0.74 | |  |
| 2.65E-01 | 31 | 0.51 | 0.52 | sym | 66 | .002 | 0.66 | |  |
| 2.18E+20 | 32 | 0.56 | 0.50 | left | 0 | < .001 | 0.80 | |  |
| 2.03E-01 | 33 | 0.55 | 0.54 | sym | 37 | .012 | 0.81 | |  |
| 1.22E+15 | 38 | 0.51 | 0.57 | right | 100 | < .001 | 0.85 | |  |
| 5.34E+04 | 45 | 0.50 | 0.52 | right | 93 | < .001 | 0.62 | |  |
| 4.06E-01 | 46 | 0.52 | 0.53 | sym | 70 | < .001 | 0.75 | |  |
| 9.67E+01 | 48 | 0.51 | 0.50 | left | 12 | < .001 | 0.67 | |  |
| 4.98E+00 | 49 | 0.51 | 0.50 | sym | 15 | < .001 | 0.72 | |  |
| 1.91E-01 | 51 | 0.54 | 0.55 | sym | 66 | .002 | 0.79 | |  |
| 5.78E-01 | 53 | 0.52 | 0.51 | sym | 22 | < .001 | 0.76 | |  |
| 8.49E-01 | 54 | 0.50 | 0.49 | sym | 33 | < .001 | 0.56 | |  |
| 1.18E+05 | 55 | 0.48 | 0.46 | left | 3 | < .001 | 0.66 | |  |
| 1.76E+02 | 61 | 0.50 | 0.48 | left | 10 | < .001 | 0.68 | |  |
| 5.29E-01 | 63 | 0.51 | 0.50 | sym | 16 | < .001 | 0.81 | |  |
| 1.74E-01 | 65 | 0.52 | 0.52 | sym | 54 | .484 | 0.73 | |  |
| 4.02E+13 | 68 | 0.57 | 0.53 | left | 1 | < .001 | 0.75 | |  |
| 6.59E+10 | 72 | 0.49 | 0.53 | right | 100 | < .001 | 0.78 | |  |
| 3.49E-01 | 75 | 0.49 | 0.50 | sym | 68 | < .001 | 0.67 | |  |
| 1.43E+17 | 84 | 0.57 | 0.52 | left | 0 | < .001 | 0.81 | |  |
| 2.53E+00 | 93 | 0.46 | 0.47 | sym | 75 | < .001 | 0.63 | |  |
| 2.17E+03 | 97 | 0.55 | 0.52 | left | 2 | < .001 | 0.80 | |  |
| 3.36E+13 | 107 | 0.56 | 0.51 | left | 0 | < .001 | 0.79 | |  |
| 1.44E+07 | 111 | 0.53 | 0.49 | left | 2 | < .001 | 0.79 | |  |
| 2.07E+04 | 113 | 0.48 | 0.50 | right | 96 | < .001 | 0.70 | |  |
| 9.94E+35 | 116 | 0.46 | 0.55 | right | 100 | < .001 | 0.86 | |  |
| 3.38E+01 | 121 | 0.50 | 0.52 | right | 90 | < .001 | 0.71 | |  |
| 1.91E-01 | 123 | 0.51 | 0.51 | sym | 56 | .271 | 0.59 | |  |
| 6.63E+06 | 125 | 0.56 | 0.54 | left | 2 | < .001 | 0.61 | |  |
| 3.97E+04 | 127 | 0.53 | 0.49 | left | 0 | < .001 | 0.81 | |  |
| 1.57E+10 | 129 | 0.52 | 0.56 | right | 100 | < .001 | 0.76 | |  |
| 1.96E+17 | 131 | 0.51 | 0.55 | right | 99 | < .001 | 0.65 | |  |
| 5.10E-01 | 132 | 0.56 | 0.57 | sym | 69 | < .001 | 0.77 | |  |
| 1.27E+04 | 134 | 0.52 | 0.50 | left | 5 | < .001 | 0.63 | |  |
| 6.41E-01 | 135 | 0.51 | 0.52 | sym | 81 | < .001 | 0.83 | |  |
| 1.70E+07 | 138 | 0.57 | 0.54 | left | 2 | < .001 | 0.78 | |  |
| 1.46E+00 | 139 | 0.50 | 0.49 | sym | 21 | < .001 | 0.67 | |  |
| 1.49E+18 | 142 | 0.50 | 0.55 | right | 100 | < .001 | 0.81 | |  |
| 4.53E+05 | 143 | 0.51 | 0.54 | right | 100 | < .001 | 0.73 | |  |
| 1.11E+00 | 144 | 0.48 | 0.49 | sym | 68 | < .001 | 0.53 | |  |
| 4.08E+01 | 146 | 0.49 | 0.51 | right | 83 | < .001 | 0.59 | |  |
| 5.59E-01 | 147 | 0.51 | 0.50 | sym | 20 | < .001 | 0.75 | |  |
| 2.11E+05 | 150 | 0.53 | 0.50 | left | 4 | < .001 | 0.71 | |  |
| 1.94E-01 | 151 | 0.51 | 0.51 | sym | 55 | .368 | 0.75 | |  |
| 6.97E+03 | 153 | 0.51 | 0.53 | right | 91 | < .001 | 0.60 | |  |
| 1.38E+07 | 154 | 0.51 | 0.56 | right | 100 | < .001 | 0.84 | |  |
| 2.60E+02 | 200 | 0.49 | 0.51 | right | 94 | < .001 | 0.72 | |  |

2. ROI Asymmetries

*bf (Bayes Factor, comparison against zero), ROI (Region of Interest), accdiff (Absolute Accuracy Difference), accdiff_se (Absolute Accuracy Difference Standard Error). Only subjects with significant asymmetry were included (compare Supplementary Table 1.A. and 1.B.).*

1. *Global Contrast Asymmetries (200ms-350ms)*

| bf | ROI | accdiff | accdiff_se |
| --- | --- | --- | --- |
| 9.80E+08 | ITG elv | 6.55 | 0.65 |
| 1.76E+10 | ITG vl | 6.52 | 0.59 |
| 7.38E+09 | V5 | 6.04 | 0.56 |
| 1.72E+11 | ITG cl | 5.69 | 0.48 |
| 1.49E+11 | FFG lv | 5.62 | 0.47 |
| 7.56E+10 | MTG dl | 5.37 | 0.46 |
| 2.98E+09 | FFG mv | 5.35 | 0.51 |
| 1.30E+09 | PHG TH | 5.32 | 0.52 |
| 2.62E+10 | IPL c | 5.28 | 0.47 |
| 3.31E+09 | STS cp | 5.08 | 0.48 |
| 1.13E+09 | STS rp | 4.90 | 0.48 |
| 1.80E+10 | MOG | 4.58 | 0.41 |
| 5.50E+12 | IPL rv | 4.57 | 0.34 |
| 7.44E+06 | IOG | 4.34 | 0.51 |
| 2.39E+10 | SOG l | 4.27 | 0.38 |
| 7.98E+10 | IPL rd | 4.19 | 0.36 |
| 1.13E+10 | LG r | 4.07 | 0.37 |
| 3.41E+11 | PC dmpos | 3.34 | 0.27 |
| 9.35E+08 | OPC | 3.10 | 0.31 |
| 2.81E+10 | MVOC vmPOS | 3.03 | 0.27 |
| 3.96E+09 | SOG m | 3.02 | 0.28 |
| 1.31E+07 | LG c | 2.70 | 0.31 |
| 1.35E+10 | CG v | 2.57 | 0.23 |
| 2.06E+10 | MVOC c | 2.10 | 0.19 |
| 3.01E+10 | MVOC r | 1.82 | 0.16 |
| 4.78E+08 | PC A31 | 1.61 | 0.16 |
| 2.30E+08 | CG d | 1.15 | 0.12 |

1. *Local Contras Asymmetries (350ms-500ms)*

| bf | ROI | accdiff | accdiff_se |
| --- | --- | --- | --- |
| 1.89E+07 | ITG vl | 4.49 | 0.41 |
| 4.99E+07 | ITG cl | 4.47 | 0.39 |
| 1.34E+07 | V5 | 4.35 | 0.40 |
| 1.54E+08 | ITG elv | 4.33 | 0.36 |
| 7.23E+09 | FFG mv | 4.27 | 0.31 |
| 3.30E+08 | STS cp | 4.23 | 0.34 |
| 3.13E+08 | IPL c | 4.12 | 0.34 |
| 9.06E+09 | IOG | 4.12 | 0.29 |
| 5.11E+06 | MOG | 3.82 | 0.37 |
| 3.38E+07 | FFG lv | 3.70 | 0.33 |
| 2.21E+06 | MTG dl | 3.56 | 0.36 |
| 2.46E+07 | TH | 3.49 | 0.32 |
| 1.54E+07 | SOG l | 3.47 | 0.32 |
| 4.85E+05 | OPC | 3.18 | 0.35 |
| 4.70E+05 | STS rp | 3.17 | 0.34 |
| 1.78E+10 | LG r | 3.13 | 0.22 |
| 1.79E+06 | IPL rv | 3.05 | 0.31 |
| 5.31E+07 | SOG m | 3.04 | 0.27 |
| 1.68E+07 | IPL rd | 3.03 | 0.28 |
| 2.65E+07 | PC dmpos | 2.75 | 0.25 |
| 1.02E+07 | CG v | 2.70 | 0.25 |
| 2.14E+08 | MVOC vmpos | 2.68 | 0.22 |
| 2.40E+06 | LG c | 2.49 | 0.25 |
| 5.99E+07 | MVOC c | 2.07 | 0.18 |
| 3.50E+07 | MVOC r | 1.89 | 0.17 |
| 1.28E+07 | PC A31 | 1.48 | 0.14 |
| 5.65E+05 | CG d | 1.23 | 0.13 |
